# An Evaluation of the Factors Affecting ‘Poacher’ Detection with Drones and the Efficacy of Machine-Learning for Detection

**DOI:** 10.3390/s21124074

**Published:** 2021-06-13

**Authors:** Katie E. Doull, Carl Chalmers, Paul Fergus, Steve Longmore, Alex K. Piel, Serge A. Wich

**Affiliations:** 1 Independent Researcher, Nottingham NG21 9FN, UK; 2 School of Computer Science, Liverpool John Moores University, Liverpool L3 3AF, UK; C.Chalmers@ljmu.ac.uk (C.C.); P.Fergus@ljmu.ac.uk (P.F.); 3 Astrophysics Research Institute, Liverpool John Moores University, Liverpool L3 3AF, UK; S.N.Longmore@ljmu.ac.uk; 4 Department of Anthropology, University College London, Taviton Street, Bloomsbury, London WC1H OBW, UK; a.piel@ucl.ac.uk; 5 School of Biological and Environmental Sciences, Liverpool John Moores University, Liverpool L3 3AF, UK; S.A.Wich@ljmu.ac.uk

**Keywords:** drones, detection, camera, TIR, RGB, poachers, canopy, time of day, angle, automated

## Abstract

Drones are being increasingly used in conservation to tackle the illegal poaching of animals. An important aspect of using drones for this purpose is establishing the technological and the environmental factors that increase the chances of success when detecting poachers. Recent studies focused on investigating these factors, and this research builds upon this as well as exploring the efficacy of machine-learning for automated detection. In an experimental setting with voluntary test subjects, various factors were tested for their effect on detection probability: camera type (visible spectrum, RGB, and thermal infrared, TIR), time of day, camera angle, canopy density, and walking/stationary test subjects. The drone footage was analysed both manually by volunteers and through automated detection software. A generalised linear model with a logit link function was used to statistically analyse the data for both types of analysis. The findings concluded that using a TIR camera improved detection probability, particularly at dawn and with a 90° camera angle. An oblique angle was more effective during RGB flights, and walking/stationary test subjects did not influence detection with both cameras. Probability of detection decreased with increasing vegetation cover. Machine-learning software had a successful detection probability of 0.558, however, it produced nearly five times more false positives than manual analysis. Manual analysis, however, produced 2.5 times more false negatives than automated detection. Despite manual analysis producing more true positive detections than automated detection in this study, the automated software gives promising, successful results, and the advantages of automated methods over manual analysis make it a promising tool with the potential to be successfully incorporated into anti-poaching strategies.

## 1. Introduction

Poaching is continually fuelling the illegal wildlife trade, and it is currently on the rise, becoming a global conservation issue [1,2]. This leads to the extinction of species, large reductions in species’ abundance, and to cascading consequences on economies, international security, and the natural world itself [3,4]. Evidence from the 2016 General Elephant Consensus (GEC) showed that one African elephant is killed every 15 min, causing their numbers to dwindle rapidly [5]. One of the main drivers of this is the increasing demand for ivory and other illegal wildlife products, particularly in Asian countries, driven by the belief that products such as rhino horn hold a significant status symbol and are relied upon in traditional medicine. Rhino horn is currently valued on the black markets in Vietnam at between USD 30,000 and 65,000 per kilogram [6,7,8].

At present, there are a number of anti-poaching techniques employed in affected countries, such as ground ranger patrols, rhino de-horning operations, community and education projects, and schemes focused on enforcing illegal wildlife trade laws [4,9]. In addition, whilst these strategies are crucial in the reduction of poaching, curbing the demand for ivory products is equally as important to ensure the long-term effectiveness of anti-poaching methods [10]. More recently, drones were considered as an addition to these methods due to their decreasing cost, higher safety compared to manned aircrafts and ranger patrols, and their flexibility to carry a variety of payloads, including high resolution cameras of different wavelengths. Drones present an opportunity for larger areas to be surveyed and controlled compared to ground-based patrols, which is useful for when ‘boots on the ground’ and other resources are limited [11,12,13].

Drones are increasingly utilised over the last few decades as a conservation tool. They are successfully used for detecting animal densities and distributions, land-use mapping, and monitoring wildlife and environmental health [14,15,16,17]. For example, Vermeulen et al. [18] used drones fitted with an RGB (red, green and blue light) camera to successfully monitor and estimate the density of elephants (*Loxodonta africana*) in southern Burkina Faso, West Africa. RGB cameras obtain images within the visible light spectrum and are generally more affordable than multispectral cameras and are found on the majority of consumer grade drones. They also obtain images with a higher resolution than multispectral cameras [13]. TIR (thermal infrared) cameras, on the other hand, operate by detecting thermal radiation emitted from objects, making them useful for detecting activity that occurs at night, such as poaching, and events in which detection of heat sources is important [13,19]. For example, studies demonstrated the use of drone-mounted thermal cameras to successfully study arboreal mammals, such as Kays et al. [20], who studied mantled howler monkeys (*Aloutta palliata*) and black-handed spider monkeys (*Ateles geoffroyi*) using this method and found thermal cameras were successful in observing troops moving amongst the dense canopy, particularly during night and early morning. A similar study by Spaan et al. [21], also studying spider monkeys (*Ateles geoffroyi*), found that, in 83% of surveys, drone-mounted thermal cameras obtained greater counts than ground surveys, which is thought to be due to the larger area able to be covered by drones.

In the last few years, the proven success of drones in conservation and the increases in wildlife crime sparked research into the use of drones to detect and reduce illegal activity such as poaching and illegal hunting [22,23,24]. However, there is a lack of studies investigating the factors that influence detection, which are instrumental for understanding the environmental situations in which poachers may elude detection as well as the technical attributes that aid successful detection. Hambrecht et al. [25] contributed to research focused on investigating this topic, and this was based on a study by Patterson et al. [12], who studied the effect of different variables on the detection of boreal caribou (*Rangifer tarandus caribou*). Hambrecht et al. [25] used similar variables and adapted the focus towards poacher detection. A number of conclusions were made from this research. Using thermal cameras significantly improved detection as well as vegetation cover having a negative impact on detection during thermal flights. Time of day was not found to be a significant factor, despite results suggesting cooler times of day improved detection. The contrast of test subjects to surroundings (e.g., the colour t-shirt they were wearing) and drone altitude significantly affected detection during RGB flights as well as canopy density.

Despite these conclusive results, there are a number of knowledge gaps which, if investigated, will provide protected area managers, NGOs, and other stakeholders with additional reliable scientific information, allowing them to make informed decisions about whether to allocate the time and the resources into incorporating drones into anti-poaching operations and how to do this in a way that maximises success. One of the knowledge gaps recognised was the effect of camera angle on poacher detection. Perroy et al. [26] conducted a study to determine how drone camera angle impacted the aerial detection of an invasive understorey plant species of miconia (*Miconia calvescens*) in Hawaii. It was found that an oblique angle significantly improved detection rate. Based on this information, this study aimed to investigate whether the same effect could be found with poacher detection. Furthermore, Hambrecht et al. [25] did not find any significance in the effect of time of day on detection, which could be due to the small number of flights that were conducted. Other studies such as one by Witczuk et al. [27] found that the timing of drone flights had a significant influence on detection probabilities depending on the camera type. It was suggested that dawn, dusk, and night-time were the optimal times for obtaining significant quality images with a thermal camera. In addition to these factors, this study aimed to investigate whether walking test subjects were more easily detected than stationary ones, particularly through TIR imaging, as walking test subjects are more easily differentiated from objects with a similar heat signature. This was also found in a study Spaan et al. [21], who successfully used drone mounted TIR cameras to detect spider monkeys (*Ateles geoffroyi*) amongst the dense canopy.

Finally, this study also tested the efficiency of a machine-learning model for automated detection using trained deep learning neural networks as an alternative to the manual analysis of collected data [28]. Thus far, research relied on data recording on-board the drone for later manual analysis, which can be labour intensive and time consuming, particularly when studying animal abundance or distribution [13,17]. Automated detection methods were successfully used previously to detect and track various species including birds and domestic animals, and they are being investigated further as a potential means for detecting threats to wildlife in real-time, e.g., poaching and illegal logging [17,29,30,31]. For example, a study by Bondi, Fang et al. [3] explored the use of drones mounted with thermal cameras and the use of an artificial intelligence (AI) application called SPOT (Systematic Poacher de-Tector) as a method for automatically detecting poachers in near real-time. It incorporated offline training of the system and subsequent online detection. More recently, a two-part study by Burke et al. [32] and Burke et al. [33] evaluated the challenges faced in automatically detecting poachers with threshold algorithms, addressing environmental effects such as thermal radiation, flying altitude, and vegetation cover on the success of automated detection. A number of recommendations were made to overcome these challenges, which are discussed and evaluated later in the paper.

This study builds upon the research by Hambrecht et al. [25], investigating the knowledge gaps as well as collecting a larger amount of data to increase the statistical power of the results. It aimed to provide additional knowledge to what was already conducted in the use of automated detection to combat wildlife crime, a field that is still in its infancy. It was predicted that, provided the deep learning model was trained sufficiently, automated detection would prove to be equally as successful in detecting poachers as manual analysis, if not more. Various studies also found this result, such as Seymour et al. [34], who used automated detection to survey two grey seal (*Halichoerus grypus*) breeding colonies in eastern Canada. Automated detection successfully identified 95–98% of human counts. In addition, it was hypothesised that, for both types of analysis, the variables having a significant effect on detection would be time of day, canopy density, and walking/stationary subjects.

## 2. Materials and Methods

### 2.1. Study Area and Flight Plan

The study took place at the Greater Mahale Ecosystem Research and Conservation (GMERC) field site in the Issa Valley, western Tanzania (latitude: −5.50, longitude: 30.56). The main type of vegetation in this region is miombo woodland, dominated by the tree genera Brachystegia and Julbernardia. This region is also characterised by a mosaic of other vegetation types such as riverine forest, swampland, and grassland [35,36]. The vegetation was dense and green due to the study being conducted in early March of 2020 towards the end of the rainy season.

An area of miombo woodland of approximately 30 × 30 m was chosen for its proximity to the field station and also for visual characteristics, as it offered a variety of canopy densities and open-canopy areas to utilise as take-off and landing zones. This site was the same approximate area in which the study by Hambrecht et al. [25] was conducted, which also influenced the choice of site, as it offered the opportunity for standardisation and to build upon the research.

Within the study area, five different sequences of locations were selected. Each sequence consisted of 4 locations, marked with blue tape, representing open, low, medium, and high canopy densities. These sequences were changed each day to create a larger sample size. Therefore, over the 7 days of data collection, a total of 35 different sequences were used, and the GNSS coordinates and the canopy density of each location were recorded. A diagram of the study site and example sequences is shown in Appendix A. A total of 20 drone flights were conducted over 7 days, with 3 flights per day at dawn (7:00), midday (13:00–13:30), and dusk (19:15). All flights conducted at dawn and dusk were conducted with a drone-mounted TIR camera, and all midday flights were conducted with a drone-mounted RGB camera. One midday flight did not proceed due to rain. The drone was hovered consistently at an altitude of 50 m for all but 2 thermal flights (which were conducted at 70 m) and in approximately the same location above the study site for each flight.

### 2.2. Drones and Cameras

The drones used for this study were a DJI Mavic Enterprise with an RGB camera for the midday studies and a DJI Inspire 1 with a FLIR Zenmuse XT camera (focal length: 6.8 mm, resolution: 336 × 256) for the TIR studies. A TIR camera was not used at midday due to the high thermal contrast at that particular time of day, and it is already known that the surrounding temperatures would significantly hinder the chances of detection [20]. The cameras mounted to both drones were continuously recording footage throughout the flights, which lasted for a maximum time of 10 min. All flights were conducted by KD and SAW.

### 2.3. Canopy Density

The canopy density of each location was classified by first taking photos of the canopy using a Nikon Coolpix P520 camera with a NIKKOR lens (focal length: 4.3–180 mm) mounted onto a tripod set at a height of 1 m. The camera was mounted at a 90° angle so that the camera lens was facing directly upwards. The photos were then converted to a monochrome BMP format using Microsoft Paint.

Following this, the canopy densities were calculated in ImageJ software by importing each photograph and obtaining the black pixel count from the histogram analysis and converting this into a percentage. The densities were then classified into open (0–25%), low (25–50%), medium (50–75%), and high (75–100%) canopy density categories.

### 2.4. Stationary or Walking Test Subjects

Five test subjects were voluntarily recruited for each flight, and each test subject was randomly assigned to one of the five sequences of locations. Beginning at the open canopy location, the test subjects were instructed to walk between each location on command, remaining stationary at each location for 10 s. They would then repeat this same routine backwards, starting at the high canopy density location and finishing at the open canopy location. See Appendix A again for a visual explanation of this. Ethical approval reference: 20/NSP/010.

### 2.5. Camera Angle

During each flight, the camera was first placed at a 90° angle, during which time the test subjects walked from the open to high canopy density. The camera was then tilted to 45°, and the drone was moved slightly off-centre from the study site for the second half of the study, where the test subjects walked from the high to the open canopy densities. The camera angle was changed via the drone’s remote controller, which had a tablet attached, giving a first-person view (FPV) of the camera as well as a scale of camera angle, allowing the pilot to adjust this remotely when required.

### 2.6. Image Processing and Manual Analysis

The drone footage for each flight was recorded in one continuous video. Each video was split into sections, representing the conditions of the flight. For example, one video was split into 14 smaller videos, 7 videos for the first half of the flight (90° camera angle) and 7 videos for the second half on the flight (45° camera angle). The 7 videos from each half represented when the test subjects were stationary (4 different canopy densities) and when they were walking between points (3 point-to-point walks).

Each video was then converted into JPG images of the same resolution using an online conversion website: https://www.onlineconverter.com/ (accessed 15 July 202). Due to the high volume of images produced per video, they were condensed down to 5 images per video, leaving 1400 overall to be analysed. The images were then split amongst 5 voluntary analysts, all of whom had never seen the images before and had no previous knowledge of the research or any experience conducting this type of analysis. Each analyst received 1 of the 5 images per video, meaning each individual was given 280 images in total to analyse. The images were presented in a random order and in controlled stages (i.e., 20 per day), and the analysts were not told how many test subjects were in the image; they simply confirmed the number of subjects they could see, which was recorded along with false positives and false negatives. In this study, false negatives were classed as subjects that were identifiable in images with a trained eye but were missed during analysis.

### 2.7. Automated Detection Software

Prior to the study, a machine-learning model was trained using a Faster-Region-based Convolutional Neural Network (Faster-RCNN) and transfer learning [37]. The training was done by tagging approximately 6000 aerial-view images of people, cars, and African animal species (elephants, rhinos, etc.), both TIR and RGB images, via the framework: www.conservationai.co.uk (accessed 9 April 2020) using the Visual Object Tagging Tool (VoTT) version 1.7.0. In order to classify objects within new images, the deep neural network extracts and ‘learns’ various parameters from these labelled images [28,38].

Following the training of the model, the drone images used for manual analysis were uploaded into the model for testing, 1400 in total. The developed algorithm subsequently analysed the characteristics within each image, also comparing them to previously tagged images, enabling positive identifications of test subjects to be automatically labelled, giving the results of automated detection [29].

### 2.8. Rock Density vs. False Positives

In addition to the core analysis of this study, the data were analysed further to establish whether more false positives occurred in the automated detection data images with a higher rock density. All 1400 images were split into three categories of rock density: low (0–40% ground cover), medium (40–70%), and high (>70%). The number of false positives in each image was recorded, and due to the number of images per category differing, the total number of detections was also recorded in order to calculate a percentage of false positives. To statistically compare the three categories, a three-proportion Z-test was conducted.

### 2.9. Statistical Analysis

All statistical analyses were carried out in R Studio using glm2, MuMIn, and ggplot2 packages [39,40]. The statistical analyses explained were repeated for both manual and automated detection data. Any data entries that contained missing values were removed from the data set (15 entries out of 7001 were excluded). The data set was split into two separate base data models, representing TIR flight data and RGB flight data. The variables used for statistical analysis are shown in Table 1. Due to the test subjects transitioning between canopy density classes when walking from point to point, 6 more factors of canopy density were added in addition to ‘open’, ‘low’, ‘med’, and ‘high’ for stationary subjects. These represented canopy density with walking subjects at a 90° camera angle (open-low, low-med, med-high) and at a 45° camera angle (high-med, med-low, low-open). The ‘open’ canopy density category was used as the reference factor for all canopy density analyses in R, using the relevel() function [41]. Time of day was not included in analysis of the RGB data model, as RGB flights were only conducted at midday. Analyst was not included as a random intercept due to the controlled environment in which the analysis took place and the fact that all analysts had no prior experience.

As the response variable was binary (i.e., detected(1)/notdetected(0)), a global generalised linear model with a logit link function was created for both RGB and thermal data [42,43]. This was done using the glm() function of the glm2 package [40]. Following the methods described by Grueber et al. [44], sub-models were created for both global models using the dredge() function from the MuMIn package [40]. This produced a list of models with every possible combination of predictor variable, along with the Akaike Information Criterion corrected for small samples (AICc), Akaike weight, log-likelihood (LogLik), and delta. For both manual and automated analysis, a total of 8 sub-models were produced for the RGB data and 16 sub-models for the TIR data. The AICc and the weight allowed for the sub-models to be compared, as a lower AICc value describes a better fit of the data, and a high Akaike weight indicates a better parsimonious fit overall [12]. To select the sub-models with the best fit to the data, the get.models() function with a cut-off value of 2AICc from the MuMIn package was used. This test ranks the sub-models by their AICc values and their Akaike weight [44,45]. The best fitting model was then tested using a generalised linear model with a logit link function, producing beta coefficient estimates and a *p*-value derived from a Wald chi-square test for each predictor variable [46]. The 95% confidence intervals were also calculated for each variable in the best-fitting model. RGB and TIR detection data were then compared using a Wald chi-square test.

For further analysis, camera angle data were incorporated into canopy density analysis for both camera type, to test whether detection probabilities for varying canopy densities differed with both camera angles.

## 3. Results

### 3.1. Manual Analysis: Thermal Data Model

**Figure 1:**
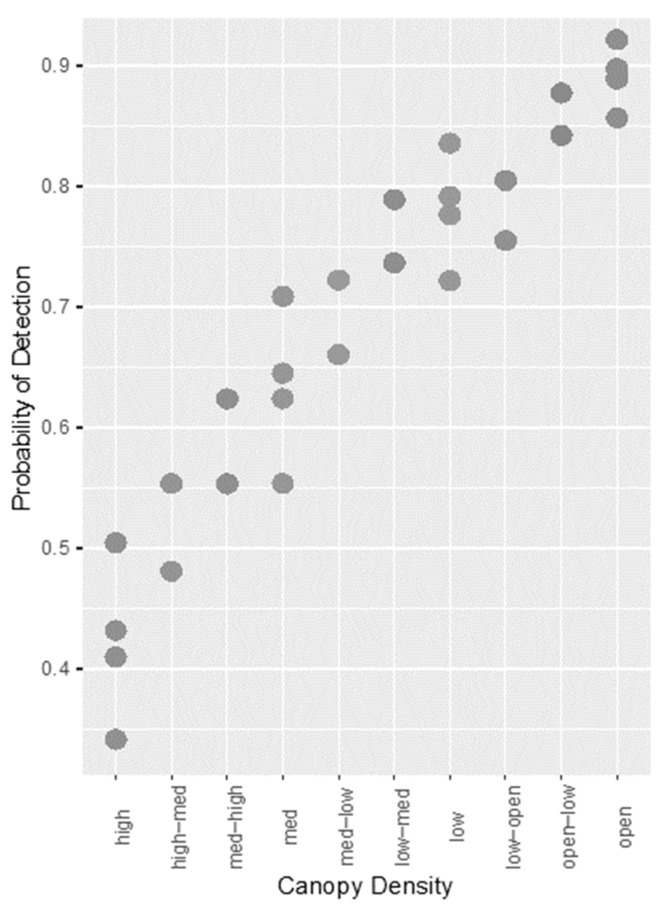
Scatterplot showing the relationship between increasing canopy density and probability of detection for TIR images with manual detection.

Through sub-model creation and selection, two models were found to offer the best fit to the data. These are shown in Appendix B, Table A1. These models had the same AICc and weight, only differing in the inclusion of walking/stationary subjects, suggesting this variable did not alter the fit of the model in any way. A Wald chi-square test was conducted to confirm this, and the variable did not significantly affect probability of detection (*p* = 0.211). Therefore, the variable was removed from the model, and the best fitting model contained the variables camera angle, canopy density, and time of day. This model is described in more detail in Table 2. Time of day was a significant factor affecting detection, indicating that there is a decreased probability of detection at dusk compared with dawn. In addition, a 90° camera angle increased probability of detection compared to an oblique angle, and an increase in canopy density caused a decrease in the probability of detection. This is portrayed in Figure 1.

For analysis of canopy density at different camera angles, a 90° angle provided a higher probability of detection at higher canopy densities than an oblique angle. This is shown in Appendix C, Table A3 in the coefficients. The number of false positives in the thermal data was 168, accounting for 74.34% of all false positives for manual analysis. Examples of these false positives are shown in Appendix D. The number of false negatives was 126, accounting for 38.06% of all false negatives for manual analysis.

### 3.2. Manual Analysis: RGB Data Model

**Figure 2:**
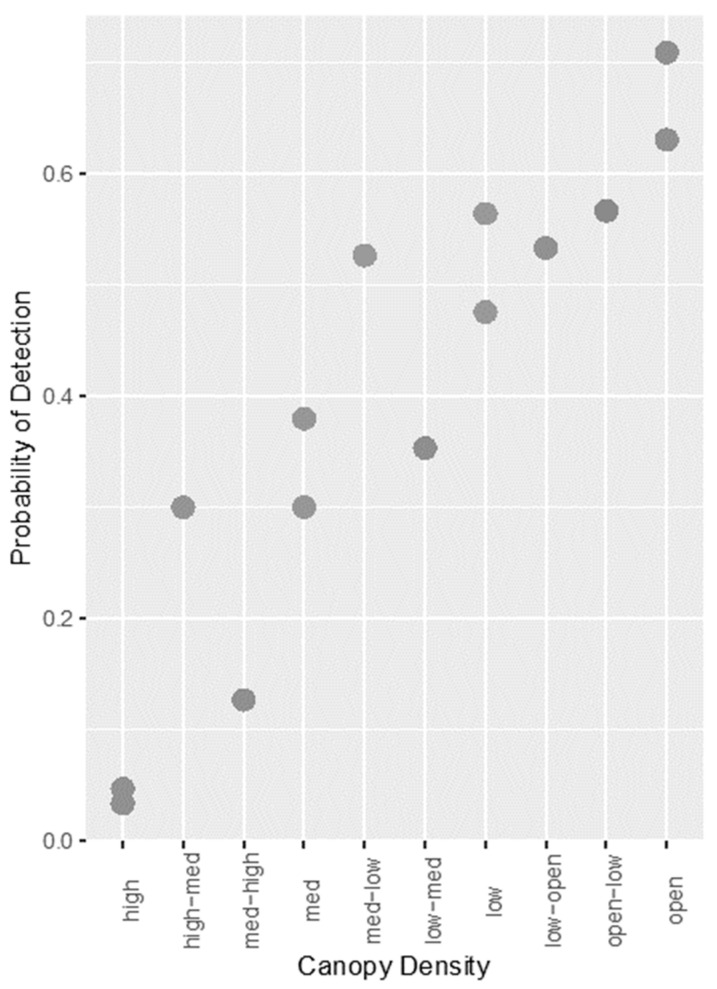
Scatterplot showing the relationship between increasing canopy density and probability of detection for RGB images with manual detection.

Two models offered the best fit to the data and are shown in Appendix B, Table A2. As with the thermal data, these two models had the same AICc and weight, again indicating that walking/stationary subjects was a redundant variable. A Wald chi-squared test confirmed this (*p* = 0.69), and the variable was removed from the model. The best fitting model contained only two predictor variables: camera angle and canopy density, and this is described in Table 3. Camera angle was a significant factor affecting detection, with a 90° angle decreasing the probability of detection compared with an oblique angle. As with the thermal data, higher canopy densities produced a significant decrease in the probability of detection, except for ‘open-low’ density (Figure 2).

**Figure 3:**
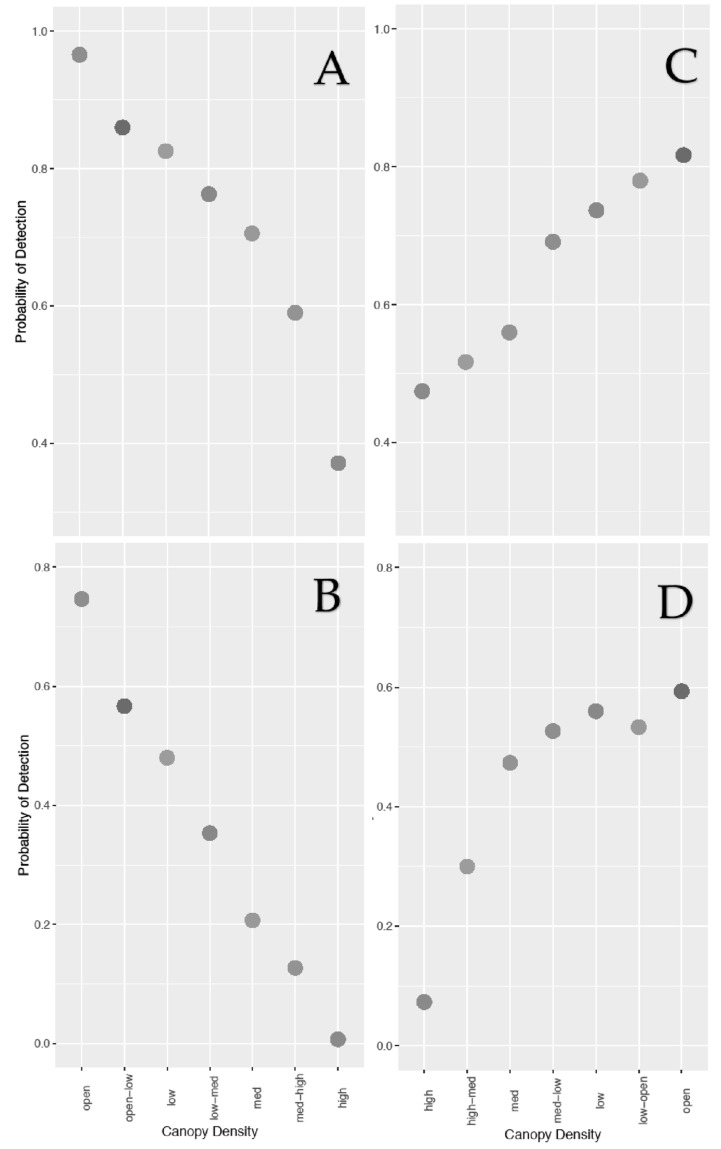
Scatterplots showing the relationship between increasing canopy density and probability of detection for both camera angles with manual detection. (**A**) Canopy vs. 90° camera angle for TIR data, (**B**) canopy vs. 90° camera angle for RGB data, (**C**) canopy vs. 45° angle for TIR data, (**D**) canopy vs. 45° angle for RGB data. The x axes for (**A**) and (**B**) (90° angle) and (**C**) and (**D**) (45° angle) are in opposite directions to represent the direction the test subject walked from point to point.

For analysis of canopy density at different camera angles, the same was found for RGB data—a 90° angle produced a higher probability of detection at high canopy densities. This relationship is shown in Figure 3 and is also described in more detail in Appendix C, Table A4. The number of false positives in the RGB data was 58, accounting for 25.66% of all false positives for manual analysis (see Appendix D). The number of false negatives was 205, accounting for 61.93% of all false positives for manual analysis.

### 3.3. Manual Analysis: Comparison between Thermal and RGB Models

The overall probability of detection was higher for thermal images (0.69, sample size: 4885) than for RGB images (0.396, sample size: 2100). The Wald chi-square test indicated a significance in the increased probability of detection with a thermal camera (*p* ≤ 2 × 10^−16^).

### 3.4. Automatic Detection Analysis: Thermal Data Model

**Figure 4:**
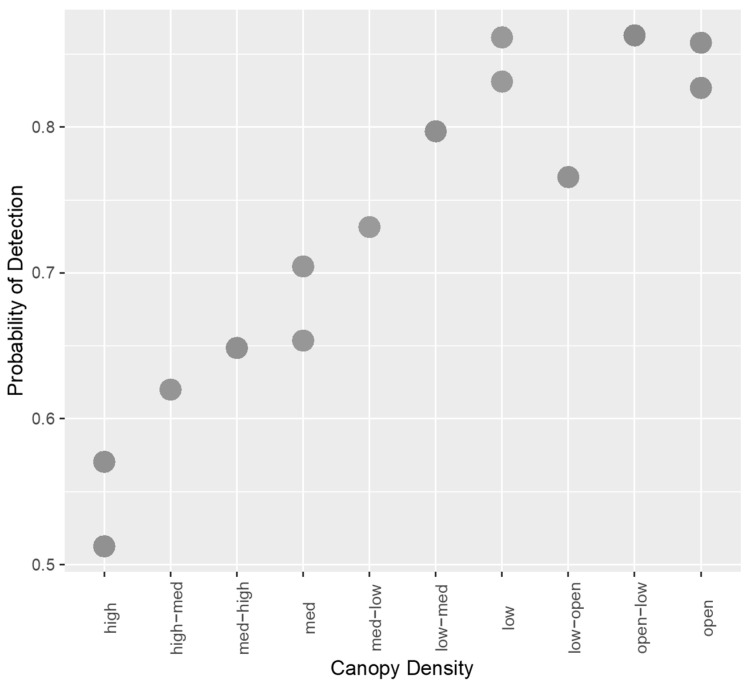
Scatterplot showing the relationship between increasing canopy density and probability of detection for TIR images with automated detection.

Sub-model creation and selection produced four models which provided the best fit to the data, and these are described in Appendix E, Table A5. Of these four, two models shared the lowest AICc and the highest Akaike weight. Similar to results from manual analysis, these two models only differed in the inclusion of walking/stationary subjects, suggesting it did not improve the model in any way, which was confirmed by the Wald chi-squared test (*p* = 0.844). The two best-fitting models also did not include time of day, and this variable was found not to be a significant contributor to the model (*p* = 0.849). Therefore, the best fitting model included the variables camera angle and canopy density. This model is described in more detail in Table 4. A 90° camera angle significantly improved detection probability, and excluding ‘low’ and ‘open-low’ densities as well as increasing canopy density significantly decreased detection probability (Figure 4). The number of false positives for the TIR data was 1059, accounting for 96.97% of all false positives for automated detection (see Appendix F). The number of false negatives was 42, accounting for 31.82% of all false negatives for automated detection.

### 3.5. Automated Detection Analysis: RGB Data Model

**Figure 5:**
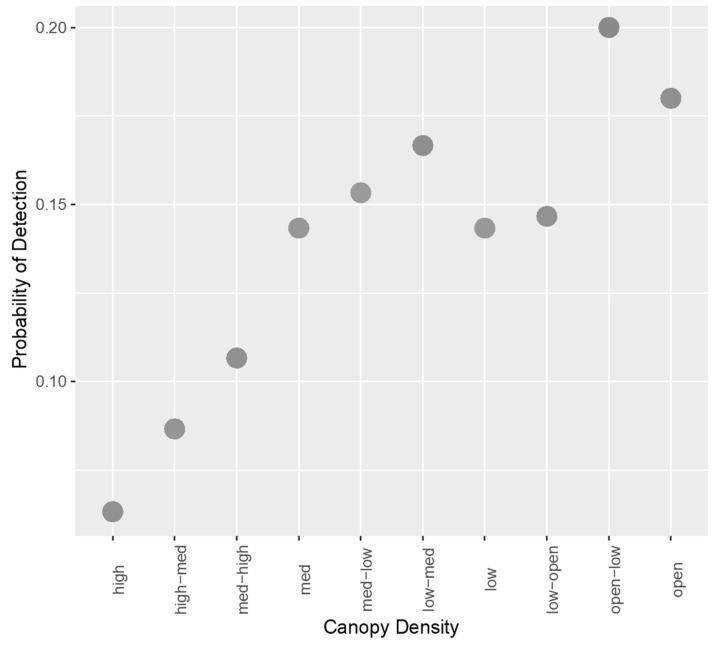
Scatterplot showing the relationship between increasing canopy density and probability of detection for RGB images with automated detection.

The four models offering the best fit to the data are shown in Appendix E, Table A6. The best fitting model included only canopy density and is described in Table 5. Camera angle (*p* = 0.704) and stationary/walking test subjects (*p* = 0.475) had no significant effect on probability of detection with an RGB camera. Increasing canopy density caused a decrease in detection probability (Figure 5), however, only three density classes were significant (high, med-high, and high-med). The number of false positives for RGB data was 33, accounting for 3.03% of all false positives for automated detection. The number of false negatives was 90, accounting for 68.18% of all false negatives for automated detection.

### 3.6. Automated Detection Analysis: Comparison between Thermal and RGB Models

The overall probability of detection was higher for thermal images (0.731, sample size: 4885) than for RGB images (0.137, sample size: 2100). The Wald chi-square test indicated a significance in the increased probability of detection with a thermal camera (*p* ≤ 2 × 10^−16^).

### 3.7. Comparison between Manual and Automated Analysis

Analysis of the variation in the number of positive detections revealed that both forms of analysis showed similar probabilities of detection (automated: 4007 positive detections, manual: 4205 positive detections), however, overall manual analysis was found to be statistically better at detecting subjects (*p* = 9.26 × 10^−8^). The number of false positives was also significantly higher with automated detection (1089) compared with manual detection (226). In contrast, the number of false negatives was higher with manual detection (331) than with automated detection (132).

Manual analysis required approximately 35 h of image analysis and recording of results, whereas the automated detection software allowed all 1400 images to be analysed within a few minutes. Recording the results of this automated detection required no more than 2 h to complete.

### 3.8. Rock Density vs. False Positives in Automated Analysis

Upon analysing each density category separately, it was found that, in images with the highest density of rocks, there was a much larger number of false positives. These results are outlined in Table 6. The results of the three-proportion Z-test revealed that the differences between the three density categories in the number of false positives were significant (*p* = 2.2 × 10^−16^).

## 4. Discussion

The aim of this study was to identify the variables that significantly affect the probability of ‘poacher’ detection using drones fitted with either a TIR or RGB camera, building upon findings from studies already conducted on this topic, in particular the study by Hambrecht et al. [25]. The factors found to have a significant effect on detection through manual analysis were time of day, camera type, camera angle, and canopy density. Walking or stationary test subjects had no significant effect on detection. Through automated analysis, camera type, camera angle, and canopy density significantly influenced detection. Time of day and walking or stationary test subjects had no significant effect. Analyst was not included as a predictor variable because, similarly to Patterson et al. [12], analysts had no previous experience with this type of analysis, and it took place in a controlled environment (i.e., a set number of images were analysed per day).

For the purpose of analysis, the drone footage was viewed through a limited number of images after the flight, but in a realistic and ideal scenario, drone footage would be analysed or monitored through a real-time video stream. Despite this, the study provides detailed information to stakeholders about the environmental and the technological factors that enhance poacher detection, such as when to fly or what camera angle to use, and also provides results on the detection capabilities of machine learning models under these different optimal conditions. At present, many studies are exploring the effectiveness of drones in more in situ scenarios and in some cases are focused on the use of real-time footage in combination with automated object detection [3,37,47,48].

### 4.1. Technical and Environmental Attributes

Overall, it was found that the probability of detection was significantly higher in flights with a TIR camera than an RGB camera. A TIR camera was not used at midday in this study; it is already known that thermal capabilities are very low during the heat of the day, as thermal contrasts are too high to distinguish features within the environment [21]. Similarly, an RGB camera was not used for dawn and dusk studies, as it relies on the presence of sunlight and thus is not useful during darker times of day [32,49]. These results thus indicate that, for detection of humans, flying a drone with thermal capabilities is likely to be more successful than flying a drone with only RGB capability.

For thermal studies with manual analysis, the probability of detection at dawn was found to be significantly higher than at dusk. The advantage of using TIR cameras is that they offer a defined thermal contrast which displays homeothermic animals, including humans, as a white object against a black background due to thermoregulatory behaviours of the individual. The thermal characteristics of the environment change throughout the day, e.g., rocks and trees heat up, giving the test subjects a reduced thermal contrast to their surroundings. Therefore, at dusk, the test subjects may have been less likely to be seen within images or easily confused amongst rocks, trees, and paths that also appear white in images [27,32,50]. This was reflected in the number of false positives found in the manual thermal data (74.34%) compared with RGB data (25.66%).

In the studies previously mentioned by Burke et al. [32,33], it was reported that the main causes of false positives were hot rocks, reflective branches, and hot or reflective patches of ground, and the best time of day to operate in order to minimise these false positives is in the early morning. It was suggested that modelling the terrain prior to drone flights could allow spurious sources to be pre-empted and discarded; also suggested was using an oblique angle to detect objects under dense vegetation.

Despite the dusk flights in this study being conducted after sunset at 19:15, at this time, it was not completely dark, and it is likely that the environmental temperatures were still high from high temperatures during the day, which subsequently had an effect on thermal contrast and detection probabilities. Despite the results of automated analysis producing a detection probability greater than 0.5, it revealed a higher number of false positives compared with manual analysis.

The number of rocks present in the thermal images were found to significantly affect these numbers of false positives that were identified in automated analysis, which was expected. In order to limit these false positives and for the model to accurately distinguish between people and aspects of the environment, the model requires more thorough training and testing before being used for operational purposes as well as incorporating some of the recommendations by Burke et al. [32,33]. In contrast, the number of false negatives was found to be higher with manual analysis compared with automated detection. These false negatives occurred due to confusion with other objects in the environment that were close by (e.g., hot rocks), vegetation cover blocking part of the subject, and a reduced thermal contrast. This result was also found in the study by Burke et al. [32].

For future improvements, it would be useful to incorporate the daily temperature recordings from the field station to observe whether this correlates with an increase or a decrease in detection probabilities for thermal flights. It would also be beneficial to conduct dusk flights later in the evening, when the environmental temperatures may be cooler, but it would be expected that detection probabilities would remain higher at dawn than dusk. Our results, however, still coincide with evidence on the modus operandi of poachers in relation to the time of day they are most likely to operate. A study by Koen et al. [19], in which an ecological model was produced to structure the rhino poaching problem, reported that most poaching events occurred at twilight, i.e., dawn and dusk. This provides a co-occurrence between the optimal time of day for drone detection and the preferred operational time for poachers.

Our study also showed a variance in detection probabilities with different camera angles. For thermal data in both forms of analysis, a 90° angle was found to increase the probability of detection, yet for RGB data (manual analysis), the opposite was true; an oblique angle gave a higher probability of detection. It was expected that, for both camera types, an oblique angle would allow for test subjects underneath the dense canopy to be more easily detected. Oblique camera angles allow for blind spots, hard-to-see environmental characteristics, and various fields of view to be exposed, none of which are facilitated by a nadir camera-view [27,33,51]. During thermal flights, when the camera was at an oblique angle, test subjects may have blended into their environment, particularly during dusk flights, being blocked by or mistaken for tree trunks or boulders by having a reduced thermal contrast. At a 90° angle, only leaves and small branches blocked the test subjects. Leaves do not absorb and retain as much heat as the trunk and cool down via transpiration, meaning, through thermal imaging, test subjects have a much higher thermal contrast compared to the leaves, making them more visible through gaps in the canopy [52]. For the RGB flights, without the advantage of a thermal contrast, it is more difficult to detect subjects, particularly in high density foliage and from 50 m above ground level (AGL). It is evident that, with an oblique camera angle, where, in thermal studies, tree trunks and test subjects were sometimes indistinguishable, subjects were more easily detected, particularly if they were wearing brighter coloured t-shirts, giving them a greater contrast against the environment (Appendix G). Similar effects were also found in other studies with both humans and animals [12,22,25].

Camera angle was not found to be a significant variable with automated analysis. Burke et al. [33] concluded that using an oblique camera angle could hinder the efficiency of automated detection, as the apparent size of objects in the camera’s field-of-view (FOV) differs due to the FOV covering a wider area at the top and becoming increasingly narrow towards the bottom. It was recommended that, to achieve the minimum resolution required to detect and identify an object with an oblique camera angle, it is necessary to reduce the altitude of the drone. This would not be beneficial in anti-poaching operations, where it is essential for the drone to remain undetected (discussed later in the paper). It is also possible the lack of significance was due to the small number of positive detections produced by the model for RGB data (288 out of 2100 potential detections). An improvement to the study would be to conduct flights where the drone is flown around the test area rather than stationary, as it could increase chances of detection by facilitating different fields of view, particularly at higher canopy densities. It would also more closely represent a real-time anti-poaching operation in which drones are deployed to survey large protected areas or known poaching hotspots [23,47].

Canopy density had a significant negative effective on probability of detection for both types of analysis. This result was also found in a variety of other studies investigating detection rates of plants, animals, and humans [23,25,26,53]. Schlossberg et al. [54] found that the detectability of African elephants (*Loxodonta africana*) was influenced by habitat type and corresponding vegetation density. It was thought that an increase in the poaching of elephants caused an increase in woody vegetation that was previously grazed by these megaherbivores, which subsequently contributed to the decrease in detectability of poachers who utilised this habitat to evade detection.

In our study, it was also found that, at higher canopy densities, a 90° camera angle was more efficient at detecting test subjects with both camera types. As already explained, with an oblique camera angle, a high density of both tree trunks and foliage makes it more difficult for subjects to be seen [26].

Walking or stationary test subjects did not affect detection for either form of analysis. It was predicted that walking subjects would be more easily detected, particularly at higher canopy densities, as they would be spotted through gaps in the canopy [20,28]. It is possible this relationship was not found due to all the flights being viewed through a limited number of photos rather than videos; if the flights were viewed as videos, it is more likely the walking subjects would be spotted. Therefore, in the future, it would be useful to analyse more photos per flight or to analyse the footage as videos as well as photos, allowing this variable to be tested under more realistic conditions [3,11]. Detection of moving subjects is made even more difficult when the camera itself is also moving (on-board the drone), but there are studies that successfully tested algorithms and detection methods that specifically track and detect moving objects with drone-mounted cameras [13,48].

### 4.2. Manual vs. Automated Detection

Automated detection software proved to be successful and time efficient in detecting subjects from both thermal and RGB drone images, despite manual analysis being statistically more advantageous. Overall, automated detection is a more convenient method to analyse data because, as previously mentioned, manual analysis is extremely error-prone and often time-consuming when large data sets need to be processed [13,50]. As shown from our results, automated detection saves a substantial amount of time spent analysing data, enabling focus to be on other important tasks, and it allows more data to be collected and analysed in a shorter timeframe. It also significantly reduces associated monetary and time costs by eliminating the need to sustain teams of researchers or rely on citizen scientists for the purpose of analysis [55,56]. For example, Norouzzadeh et al. [57] explored the use of deep learning to automatically detect 48 species of wildlife in 3.2 million camera trap images and found that automated detection saved researchers >8.2 years of human analysis of this data set. It also opens doors to a wider range of other conservation-related uses, such as real-time monitoring of environmental health, monitoring the expansion of invasive species, and allowing larger-scale projects to be more practical and feasible [29,55,58].

In the context of this research, automated detection systems mounted on-board drones can aid in increasing the speed and the efficiency at which poachers are prevented and detained. Previously, anti-poaching operations with incorporated drones relied on individuals continually supervising a live video stream, during which the detection of a poacher is then communicated with ground patrols. This method relies on the reaction time of those involved, which can be subject to human error, distraction, and delay, particularly if more than one drone is deployed at the same time. A delay of just 5 min is long enough for a rhino to be killed and de-horned. With automated detection, masses of incoming data can be quickly filtered and analysed, instantly notifying ground patrols at the presence of a poacher, who can produce quicker responses [7,11,58]. Including the system used in this study, automated detection systems are also using more complex deep learning pipelines and Faster-RCNNs to improve the speed at which poachers are detected. Bondi, Kapoor et al. [59] used a Faster-RCNN to detect poachers in near real-time from drones and found the inference time of the Faster-RCNN was 5 frames per second (fps), whilst that of the live video stream was 25 fps, meaning the accuracy and the synchronisation of the detection pipeline were compromised. Therefore, a downside of this method is that the training and the use of these models in near real-time are extremely costly, along with a steep learning curve, due to the complex network and computational requirements. However, research is currently being conducted to overcome these challenges [38,60].

Whilst there are technological approaches other than deep learning for automating the detection of objects, such as edge detection, they often rely on experts manually defining and fine-tuning parameters for specific drone models and cameras before each mission. Guirado et al. [61] evaluated the success of machine learning for detecting and mapping *Ziziphus lotus* shrubs compared with the more commonly used object-based image analysis (OBIA). The deep learning method achieved 12% better precision and 30% better recall than OBIA. Deep neural networks within these deep learning models have the ability to extract parameters directly from labelled examples as a result of supervised learning [3,57].

### 4.3. Challenges for the Usage of Drones in Conservation Management

Wildlife protection and conservation efforts require adaptation of methods and resources in order to keep up with the continual growth of international trade networks and the detriment caused to wildlife numbers and the environment [47].

Manned aircrafts are widely used in anti-poaching operations and other conservation-related applications such as mapping land cover change and monitoring animal distributions [13,62,63]. However, lower implementation costs, high spatial and temporal resolution of data, and higher safety of drones deem them a more feasible option for these conservation purposes. The facilitation of high-resolution data collection allowed for evidence of poaching and logging, such as fire plumes from poaching camps, to be detected from as high as 200 m above ground level [64,65]. In addition, the lower noise emissions and the ability to fly lower than manned aircrafts are extremely beneficial for anti-poaching, as the drone can remain undetected whilst offering a higher chance of detecting poaching activity. This reduced noise compared with manned aircrafts is also beneficial in reducing negative impacts on wildlife and the environment and is a less invasive technique for tracking and monitoring small populations of endangered species [58,66]. A study compared the noise levels of drones and manned aircrafts, during which the flying altitude was standardised at 100 m. At this altitude, a fixed-wing drone produced 55 dBA, whereas the manned aircraft produced 95 dBA [67].

Drones are also capable of monitoring hostile or otherwise inaccessible environments (e.g., montane forests, dense mangroves, swamps), some of which may be unsociable territory for ground-based patrols [68]. Other positive social implications of using drones in conservation include empowerment and strengthening of local communities, enabling them to take conservation efforts into their own hands by collecting their own data and helping to raise awareness of conservation issues amongst the community. This is made possible due to the high-resolution images and the large amount of data they can provide [68,69]. On the other hand, the presence of drones within community areas may invoke confusion and hostility if residents do not understand the purpose of the drone, particularly in developing countries where exposure to modern technology is limited. However, drones can also have an indirect positive effect on feelings of safety within communities if these drones reduce the activity of local criminals and military forces [69].

Despite the obvious advantages of drones over alternative methods, there are still drawbacks that should be considered to maximise the success of drones as an anti-poaching tool. Even though they have the ability to cover more ground for surveillance than ground patrols, the small size of most drones means they have a limited payload capacity, restricting the size of battery they can carry. The areas required to be surveyed are often extremely large, such as Kruger National Park (19,500 km^2^), and due to battery restrictions, the maximum flight time is around 1 h at optimal speed, giving the drone a footprint that covers less than 1% of the park. This is particularly problematic considering poachers are continually adapting and improving their methods and operating in unpredictable ways. Studies are exploring the use of models that can predict poacher behaviour, thus drone flight plans can be optimised to increase the chances of detection [13,67,70].

In addition, there is no way as of yet to completely silence drones, and despite them being quieter and able to fly lower than manned aircrafts, in order to remain undetected by poachers, the drone must still be a significant distance AGL. This can reduce the spatial resolution of surveillance cameras on-board and could, in turn, hinder the efficiency of automated detection. This is particularly problematic when conservation strategies are using cheaper lightweight models for automated detection. A strategy called ‘You Only Look Once’ (YOLO) uses a lightweight model with a single CNN, and although this provides faster inference time, it has the downside of having a poor overall accuracy of detection. This accuracy worsens when the drone is flown at higher altitudes [38,71]. More advanced drones and software can be used to overcome these limitations, having longer operating times and higher spatial resolutions, but they are restrictive in terms of their cost, which is unrealistic for small NGOs in developing countries. Research was conducted on ways of attenuating the noise of drones through methods such as adding propellers with more blades and installing engine silencers or propeller sound absorbers. This could overcome the need to jeopardize spatial resolution by flying higher but, again, they require additional funds that may not be readily accessible [59,72]. On the other hand, rather than being a stealthy surveillance technique, drones are also found to act as a deterrent themselves if poachers are aware they are being used in the area [73,74].

Overall, drones have the potential to be a useful addition to methods already employed within anti-poaching operations. They cannot tackle poaching as a stand-alone initiative, rather, they are best incorporated in combination with other methods; they can offer additional support where other methods may be lacking, such as larger ground cover as support for ground-based patrols. Although advancements in anti-poaching technology are crucial in order to ‘keep up’ with the increased sophistication and militarisation of poaching methods, technology as a whole cannot be the sole driver of conservation efforts. Enforced legislation and community involvement are important factors attributed to a decline in poaching incidents [59,70].

## 5. Conclusions

We outlined the technological factors that can optimise poacher detection during manual and automated detection, and whilst accurate and reliable detection comes with a number of environmental challenges, we accounted for some of these issues, such as vegetation cover and thermal signatures of the environment, by providing technological recommendations to overcome them.

We discussed how drones can benefit anti-poaching efforts better than other methods, and we showed the potential of drones as the future of conservation strategies when coupled with automated detection and when these technological and environmental factors are considered. The data suggest that, overall, using a thermal camera at either dawn or dusk provides the most success in detecting poachers. However, an RGB camera is more useful during the day when environmental temperatures are higher. Poachers under high density vegetation are less likely to be detected, but detection is improved when a 90° camera angle is used at this high density. Automated detection software had a successful detection probability of greater than 0.5, but the full potential of the software could be reached through more training of the model, and this would also reduce the large number of false positives produced in detection results. All of the findings summarised in combination with findings from other studies about advancements in machine learning, poaching hotspots, and the success of drones in anti-poaching scenarios [3,23,24,25,30,48,50,54,74] will be useful when incorporating drones into anti-poaching operations and will aid in increasing the efficiency of expenditure of anti-poaching resources. In future research, it would be beneficial to repeat this study with a focus on the efficiency of automated detection in real-time video on-board the drone under technological and environmental conditions recommended by this study to increase detection probability.

## Figures and Tables

**Table 1:** A summary of the variables used for statistical analysis including their description.

No.	Variable	Variable Type	No. of Factors	Description
	Response variable			
0	Detected	Binary	2	Detected = 1, not detected = 0
	Predictor variables			
1	Time of day	Nominal	2	Dawn = 1, dusk = 3
2	Camera angle	Binary	2	90° = 1, 45° = 0
3	Walking/stationary	Binary	2	Walking = 1, stationary = 0
4	Canopy density	Nominal	10	Canopy density class, e.g., open, low, med-low, open-low, etc.

**Table 2:** The model with the best fit to the data for TIR images with manual detection includes time of day, camera angle, and canopy density.

	Estimate	95% Confidence Intervals	*p*-Value
(Intercept)	2.0822	1.828, 2.349	<2 × 10^−16^
Time of day (dusk)	−0.292	−0.422, −0.163	9.65 × 10^−6^
Camera angle (90°)	0.383	0.209, 0.558	0.00016
Canopy density (low)	−0.837	−1.141, −0.542	4.09 × 10^−8^
Canopy density (med)	−1.576	−1.865, −1.295	<2 × 10^−16^
Canopy density (high)	−2.448	−2.738, −2.169	<2 × 10^−16^
Canopy density (open to low)	−0.496	−0.892, −0.0941	0.0145
Canopy density (low to med)	−1.145	−1.504, −0.788	3.48 × 10^−10^
Canopy density (med to high)	−1.959	−2.299, −1.627	<2 × 10^−16^
Canopy density (high to med)	−1.867	−2.198, −1.544	<2 × 10^−16^
Canopy density (med to low)	−1.125	−1.466, −0.789	6.73 × 10^−11^
Canopy density (low to open)	−0.665	−1.0213, −0.308	0.000253

**Table 3:** The model with the best fit for RGB images with manual detection includes the variables camera angle and canopy density.

	Estimate	95% Confidence Intervals	*p*-Value
(Intercept)	0.891	0.615, 1.175	**4.13 × 10^−10^**
Camera angle (*90°*)	−0.356	−0.622, 0.0906	**0.00868**
Canopy density (*low*)	−0.633	−0.967, −0.303	**0.000185**
Canopy density (*med*)	−1.382	−1.726, −1.044	**1.84 × 10^−15^**
Canopy density (*high*)	−3.906	−4.582, −3.319	**<2 × 10^−16^**
Canopy density (*open to low*)	−0.268	−0.689, 0.155	**0.214**
Canopy density (*low to med*)	−1.140	−1.567, −0.713	**2.20 × 10^−7^**
Canopy density (*med to high*)	−2.467	−3.044, −1.934	**<2 × 10^−16^**
Canopy density (*high to med*)	−1.739	−2.193, −1.297	**2.58 × 10^−14^**
Canopy density (*med to low*)	−0.785	−1.212, −0.360	**0.000299b**
Canopy density (*low to open*)	−0.758	−1.185, −0.333	**0.000481**

**Table 4:** The model with the best fit to the data for TIR images with automated detection includes the variables camera angle and canopy density.

	Estimate	95% Confidence Intervals	*p*-Value
(Intercept)	1.564	1.359, 1.777	**<2 × 10^−16^**
Camera angle (*90°*)	0.234	0.0648, 0.403	**0.0068**
Canopy density (*low*)	0.0303	−0.248, 0.309	**0.831**
Canopy density (*med*)	−0.929	−1.1801, −0.682	**2.59 × 10^−13^**
Canopy density (*high*)	−1.514	−1.761, −1.272	**<2 × 10^−16^**
Canopy density (*open to low*)	0.0418	−0.328, 0.423	**0.827**
Canopy density (*low to med*)	−0.429	−0.767, −0.0861	**0.0135**
Canopy density (*med to high*)	−1.185	−1.496, −0.875	**6.66 × 10^−14^**
Canopy density (*high to med*)	−1.074	−1.376, −0.775	**2.41 × 10^−12^**
Canopy density (*med to low*)	−0.562	−0.877, −0.245	**0.000482**
Canopy density (*low to open*)	−0.379	−0.702, −0.0539	**0.0216**

**Table 5:** The model with the best fit to the data for TIR images with automated detection includes the variables camera angle and canopy density.

	Estimate	95% Confidence Intervals	*p*-Value
(Intercept)	−1.516	−1.821, −1.230	**<2 × 10^−16^**
Canopy density (*low*)	−0.271	−0.712, 0.164	**0.223**
Canopy density (*med*)	−0.271	−0.712, 0.164	**0.223**
Canopy density (*high*)	−1.177	−1.750, −0.644	**2.72 × 10^−5^**
Canopy density (*open to low*)	0.130	−0.375, 0.621	**0.608**
Canopy density (*low to med*)	−0.0931	−0.627, 0.418	**0.726**
Canopy density (*med to high*)	−0.609	−1.233, −0.0337	**0.0453**
Canopy density (*high to med*)	−0.839	−1.518, −0.227	**0.0103**
Canopy density (*med to low*)	−0.192	−0.740, 0.3296	**0.479**
Canopy density (*low to open*)	−0.245	−0.801, 0.283	**0.374**

**Table 6:** Results of automated image analysis showing the total number of false positives in each density category as well as the total number of detections and a calculated percentage of false positives.

Rock Density	No. of False Positives	Total Number of Detections	Percentage of False Positives (%)
Low	178	1578	**11.28**
Medium	429	1498	**28.64**
High	659	1152	**57.21**

**Figure A1:**
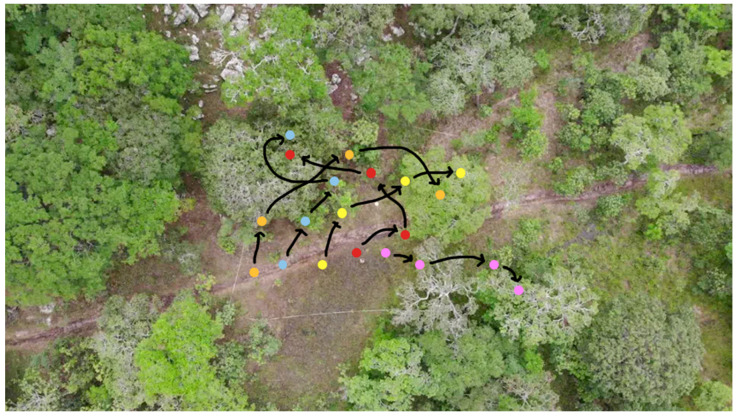
Diagram of the study site and the example locations. The white measuring tape indicates the boundaries of the study site (30 × 30 m). The different coloured spots represent the different sequences of locations. The arrows indicate where the test subject was instructed to walk; they then repeated this same walk backwards.

**Figure A2:**
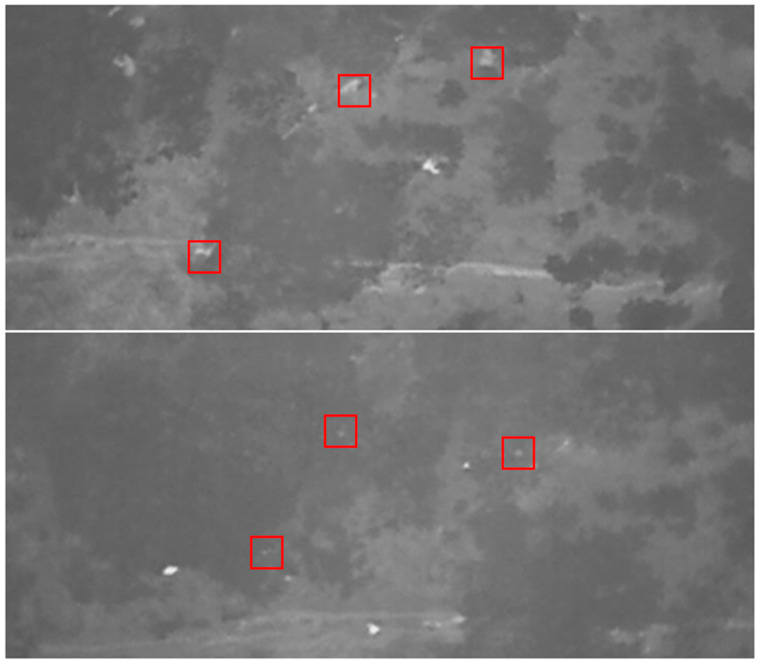
Thermal images showing the false positives caused by hot ground (top) and hot rocks (bottom). The red squares indicate the false positives.

**Figure A3:**
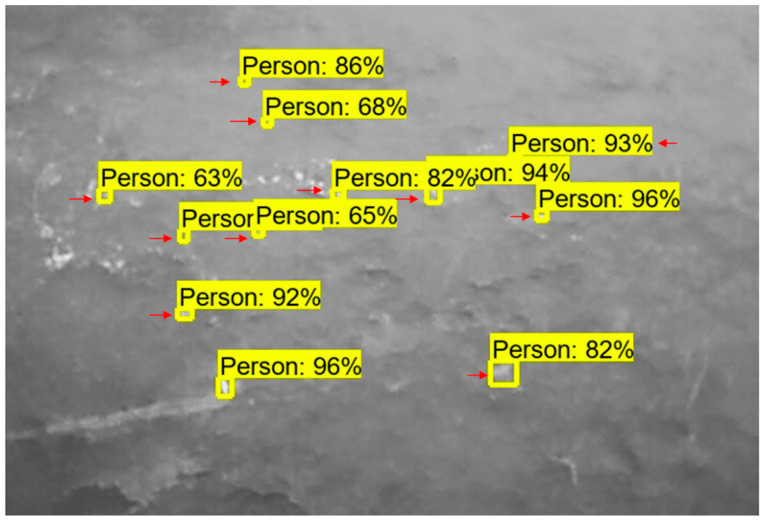
Thermal images showing the false positives caused by hot ground and rocks. The red arrows indicate the false positives.

**Figure A4:**
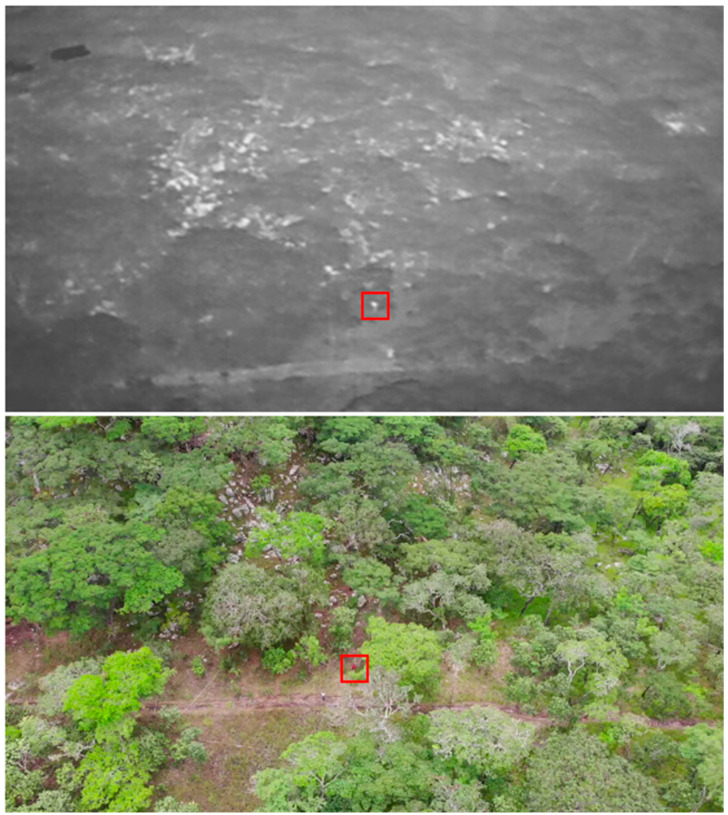
Comparison between thermal (top) and RGB images (bottom) at an oblique angle. Both images were taken in the same area and with the test subject in the same location. The red square indicates the location of the test subject.

## Data Availability

The images used for the analyses are available from the corresponding author on request as well as the R code and the datafiles.

## Appendix B. Model Selection for Manual Analysis

**Table A1:** The models with the best fit to the TIR data showing AICc and weight values.

	Df	LogLik	AICc	Delta	Weight
Camera angle + canopy density	11	−1193.274	2408.7	0.000	0.46
Camera angle + canopy density + stationary/walking	11	−1193.274	2408.7	0.000	0.46

**Table A2:** The models with the best fit to the RGB data showing AICc and weight values.

	Df	LogLik	AICc	Delta	Weight
Camera angle + canopy density + time of day	12	−2728.998	5482.1	0.000	0.5
Camera angle + canopy density + stationary/walking + time of day	16	−2728.998	5482.1	0.000	0.5

## Appendix C. Probability of Detection vs. Canopy Density at Different Camera Angles for Manual Analysis

**Table A3:** A summary of coefficients and *p*-values showing the effect of canopy density on detection with different camera angles for TIR data with manual analysis.

90° Camera Angle			45° Camera Angle		
	Estimate	*p*-Value		Estimate	*p*-Value
(Intercept)	3.338	**<2 × 10^−6^**	(Intercept)	1.497	**<2 × 10^−6^**
Canopy density			Canopy density		
*Open-low*	−1.523	**4.36 × 10^−6^**	*High*	−1.599	**<2 × 10^−6^**
*Low*	−1.783	**4.39 × 10^−8^**	*High-med*	−1.429	**3.06 × 10^−16^**
*Low-med*	−2.1697	**1.09 × 10^−11^**	*Med*	−1.256	**7.72 × 10^−13^**
*Med*	−2.464	**6.55 × 10^−15^**	*Med-low*	−0.6903	**0.000129**
*Med-high*	−2.973	**<2 × 10^−16^**	*Low*	−0.466	**0.0114**
*High*	−3.864	**<2 × 10^−16^**	*Low-open*	−0.231	**0.221**

**Table A4:** A summary of coefficients and *p*-values showing the effect of canopy density on detection with different camera angles for RGB data with manual analysis.

90° Camera Angle			45° Camera Angle		
	Estimate	*p*-Value		Estimate	*p*-Value
(Intercept)	1.0809	**8.53 × 10^−9^**	(Intercept)	0.378	**0.0230**
Canopy density			Canopy density		
*Open-low*	−0.813	**0.00114**	*High*	−2.914	**<2 × 10^−6^**
*Low*	−1.161	**3.10 × 10^−6^**	*High-med*	−1.225	**4.97 × 10^−7^**
*Low-med*	−1.685	**3.14 × 10^−11^**	*Med*	−0.485	**0.0377**
*Med*	−2.426	**<2 × 10^−16^**	*Med-low*	−0.271	**0.245**
*Med-high*	−3.0117	**<2 × 10^−16^**	*Low*	−0.137	**0.559**
*High*	−6.0849	**2.49 × 10^−9^**	*Low-open*	−0.244	**0.295**

## Appendix E. Model Selection for Automated Analysis

**Table A5:** The models with the best fit to the TIR data showing AICc and weight values.

	Df	LogLik	AICc	Delta	Weight
Camera angle + canopy density	11	−2796.136	5614.3	0.00	0.338
Camera angle + canopy density + stationary/walking	11	−2796.136	5614.3	0.00	0.338
Camera angle + canopy density + time of day	12	−2796.090	5616.2	1.92	0.130
Camera angle + canopy density + stationary/walking + time of day	12	−2796.090	5616.2	1.92	0.130

**Table A6:** The models with the best fit to the RGB data showing AICc and weight values.

	Df	LogLik	AICc	Delta	Weight
Canopy density	10	−823.393	1666.9	0.00	0.348
Canopy density + stationary/walking	10	−823.393	1666.9	0.00	0.348
Canopy density + camera angle	11	−823.213	1668.6	1.66	0.152
Canopy density + stationary/walking + camera angle	11	−823.213	1668.6	1.66	0.152
